# *cis*-Bis(azido-κ*N*)bis­(pyridine-2-carbox­amide-κ^2^ *N* ^1^,*O*)nickel(II)

**DOI:** 10.1107/S1600536808000299

**Published:** 2008-01-09

**Authors:** Marijana Đaković, Zora Popović

**Affiliations:** a Department of Chemistry, Laboratory of General and Inorganic Chemistry, Faculty of Science, University of Zagreb, Horvatovac 102a, HR-10000 Zagreb, Croatia

## Abstract

The title compound, [Ni(N_3_)_2_(C_6_H_6_N_2_O)_2_], was obtained as the first crystalline product from the reaction of Ni(NO_3_)_2_·6H_2_O, picolinamide and NaN_3_ in aqueous media. After a few days in the mother liquor, crystals of the *cis* isomer transformed into the *trans* isomer [Đaković & Popović (2007 ▶). *Acta Cryst.* C**63**, m507–m509]. The Ni atom exhibits a distorted octa­hedral environment and contains two azide ions and two planar *N*,*O*-chelating picolinamide ligands, all *cis* related. The dihedral angle between the two chelate rings is 82.43 (7)°. Pairs of mol­ecules are linked by N—H⋯N hydrogen bonds into cyclic *R*_2_^2^(16) dimers, which are further packed into a three-dimensional framework by *C*(6) and *C*(8) chains by N—H⋯N hydrogen bonds.

## Related literature

For information on the importance of azides in complexation, see Yuwen *et al.* (2000 ▶). A *trans* isomer of the title compound [Ni(N_3_)_2_(C_6_H_6_N_2_O)_2_] has been reported by Đaković & Popović (2007 ▶). For related literature, see: Allen *et al.* (1987 ▶); Bernstein *et al.* (1995 ▶); Etter (1990 ▶).
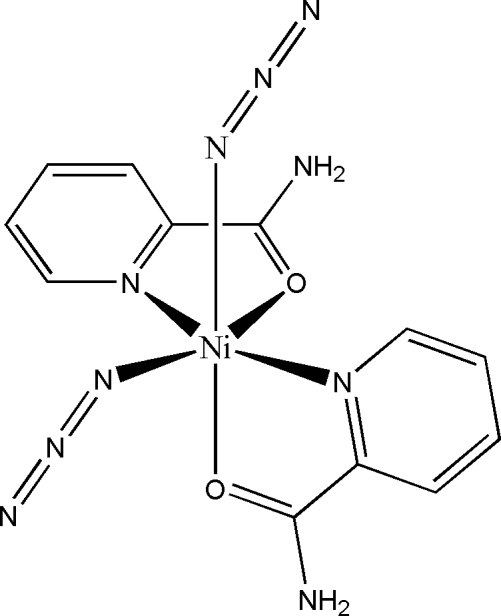


## Experimental

### 

#### Crystal data

[Ni(N_3_)_2_(C_6_H_6_N_2_O)_2_]
*M* *_r_* = 387.01
Monoclinic, 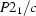

*a* = 14.3438 (5) Å
*b* = 6.6986 (2) Å
*c* = 18.7969 (10) Å
β = 120.738 (3)°
*V* = 1552.34 (12) Å^3^
*Z* = 4
Mo *K*α radiation
μ = 1.28 mm^−1^
*T* = 296 K
0.22 × 0.18 × 0.05 mm

#### Data collection

Oxford Diffraction Xcalibur diffractometer with Sapphire3 detector
Absorption correction: multi-scan (*CrysAlisPro*; Oxford Diffraction, 2007 ▶) *T*_min_ = 0.815, *T*_max_ = 0.938
15901 measured reflections
4516 independent reflections
2692 reflections with *I* > 2σ(*I*)
*R*_int_ = 0.034

#### Refinement

*R*[*F* ^2^ > 2σ(*F* ^2^)] = 0.030
*wR*(*F* ^2^) = 0.068
*S* = 0.89
4516 reflections
242 parameters
H atoms treated by a mixture of independent and constrained refinement
Δρ_max_ = 0.43 e Å^−3^
Δρ_min_ = −0.30 e Å^−3^

### 

Data collection: *CrysAlisPro* (Oxford Diffraction, 2007 ▶); cell refinement: *CrysAlisPro*; data reduction: *CrysAlisPro*; program(s) used to solve structure: *SHELXS97* (Sheldrick, 2008 ▶); program(s) used to refine structure: *SHELXL97* (Sheldrick, 2008 ▶); molecular graphics: *ORTEP-3 for Windows* (Farrugia, 1997 ▶) and *Mercury* (Macrae *et al.*, 2006 ▶); software used to prepare material for publication: *PLATON* (Spek, 2003 ▶).

## Supplementary Material

Crystal structure: contains datablocks global, I. DOI: 10.1107/S1600536808000299/kp2158sup1.cif

Structure factors: contains datablocks I. DOI: 10.1107/S1600536808000299/kp2158Isup2.hkl

Additional supplementary materials:  crystallographic information; 3D view; checkCIF report

## Figures and Tables

**Table:** 

Ni1—O1	2.0701 (13)
Ni1—O2	2.0941 (12)
Ni1—N1	2.0685 (17)
Ni1—N3	2.0559 (17)
Ni1—N5	2.0652 (14)
Ni1—N8	2.0863 (17)

**Table:** 

O1—Ni1—O2	93.30 (5)
O1—Ni1—N1	78.45 (6)
O1—Ni1—N3	89.76 (6)
O1—Ni1—N5	88.69 (6)
O1—Ni1—N8	175.91 (6)
O2—Ni1—N1	94.34 (6)
O2—Ni1—N3	78.08 (6)
O2—Ni1—N5	173.44 (7)
O2—Ni1—N8	89.94 (5)
N1—Ni1—N3	165.67 (6)
N1—Ni1—N5	92.18 (6)
N1—Ni1—N8	98.83 (7)
N3—Ni1—N5	95.69 (7)
N3—Ni1—N8	93.36 (7)
N5—Ni1—N8	88.36 (6)

**Table 2:** Hydrogen-bond geometry (Å, °)

*D*—H⋯*A*	*D*—H	H⋯*A*	*D*⋯*A*	*D*—H⋯*A*
N2—H12⋯N7^i^	0.86 (3)	2.12 (2)	2.967 (3)	165 (3)
N2—H13⋯N10^ii^	0.85 (3)	2.31 (3)	3.154 (4)	172 (2)
N4—H14⋯N8^iii^	0.80 (2)	2.36 (2)	3.136 (3)	164 (2)
N4—H15⋯N10^iv^	0.94 (3)	2.50 (3)	3.442 (4)	179 (3)

Symmetry codes: (i) 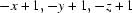; (ii) 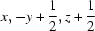; (iii) 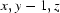; (iv) 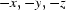.

**Fig. 1.**
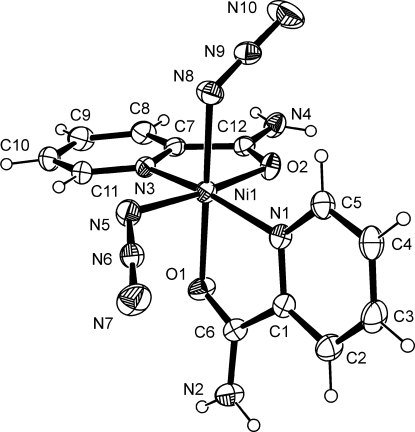
The ORTEP-3 drawing of (I) with the atom numbering scheme. Displacement ellipsoids for non-H atoms are drawn at the 30% probability level.

**Fig. 2.**
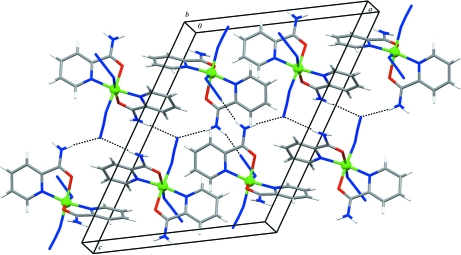
Crystal packing of (I) showing the hydrogen bonds as dashed lines.

## supplementary crystallographic information

### Comment

This research is a part of our wider interest of the structural role of azide ions and of its metal complexes in metabolic processes of mitohondria (Yuwen *et al.*, 2000).

In the title compound Ni^II^atom lies in a general position and exhibits distorted octahedral environment (Fig. 1). The coordination sphere is composed by two *cis*-related *N,O*-chelating picolinamide and two azide ligands. The picolinamide ligands are bonded more tightly (Table 1) than in its *trans*-isomer (Đaković & Popović, 2007). All other bond lengths are comparable to the values reported for similar compounds (Allen *et al.*, 1987). The azide ligands are coordinated to the central metal ion in non-linear mode (123.7 (1) and 123.0 (1)°) with the azide bond angles being 178.2 (2) and 176.9 (3)°.

The crystals of the title compound (I) are turquoise-green apart from the crystals of its *trans*-isomer which are olive-green.

The crystal structure (Fig 2) is stabilized by N—H···N hydrogen bond network between carboxamide groups and azide ligands. Typical amide N—H···O carboxamide dimers of *R*_2_^2^(8) found in *trans*-isomer are not observed in the *cis*-isomer. Instead, the amide N atoms, N2 and N4, are involved in two hydrogen bonds, forming *R*_2_^2^(16) rings, between two neighbouring molecules whereas C(8) chains along the axis c and C(6) chains along the axis b complete the network (Bernstein *et al.*, 1995; Etter, 1990).

The slightly smaller density of (I), and the fact that it is formed first and then transformed into its *trans-*isomer, suggests that (I) is the thermodinamically less stable isomer.

### Experimental

The title compound was obtained by *in sit*u reaction from Ni^II^ nitrate hexahydrate, sodium azide and picolinamide in a 1: 2: 2 molar ratio. All starting substances were dissolved in water. The sodium azide solution was added in small portions with stirring into the solution mixture of the picolinamide and Ni^II^ nitrate. In a few h the dark-green crystals of (I) were isolated. If the crystals are left in a mother liquor for a few days the dark-green crystals of (I) were transformed into the olive-green *trans*-isomer.

### Refinement

Aromatic H atoms were fixed in geometrically idealized positions and refined using a riding model with [C—H = 0.93 Å and *U*_iso_(H) = 1.2*U*_eq_(C)]. The amide H atoms were placed in the positions indicated by difference electron-density maps and their positions were allowed to refine together with individual isotropic displacement parameters.

### Crystal data

**Table:**

[Ni(N_3_)_2_(C_6_H_6_N_2_O)_2_]	*F*_000_ = 792
*M**_r_* = 387.01	*D*_x_ = 1.656 Mg m^−^^3^
Monoclinic, *P*2_1_/*c*	Mo *K*α radiation λ = 0.71073 Å
Hall symbol: -P 2ybc	Cell parameters from 5015 reflections
*a* = 14.3438 (5) Å	θ = 3.8–32.4º
*b* = 6.6986 (2) Å	µ = 1.28 mm^−^^1^
*c* = 18.7969 (10) Å	*T* = 296 K
β = 120.738 (3)º	Plate, blue
*V* = 1552.34 (12) Å^3^	0.22 × 0.18 × 0.05 mm
*Z* = 4	

### Data collection

**Table:**

Oxford Diffraction Xcalibur diffractometer with Sapphire3 detector	4516 independent reflections
Radiation source: Enhance (Mo) X-ray Source	2692 reflections with *I* > 2σ(*I*)
Monochromator: graphite	*R*_int_ = 0.034
Detector resolution: 16.3426 pixels mm^-1^	θ_max_ = 30.0º
*T* = 296 K	θ_min_ = 3.8º
CCD scans	*h* = −20→20
Absorption correction: multi-scan(CrysAlisPro; Oxford Diffraction, 2007)	*k* = −9→9
*T*_min_ = 0.815, *T*_max_ = 0.938	*l* = −26→26
15901 measured reflections	

### Refinement

**Table:**

Refinement on *F*^2^	Secondary atom site location: difference Fourier map
Least-squares matrix: full	Hydrogen site location: inferred from neighbouring sites
*R*[*F*^2^ > 2σ(*F*^2^)] = 0.030	H atoms treated by a mixture of independent and constrained refinement
*wR*(*F*^2^) = 0.068	*w* = 1/[σ^2^(*F*_o_^2^) + (0.0323*P*)^2^] where *P* = (*F*_o_^2^ + 2*F*_c_^2^)/3
*S* = 0.89	(Δ/σ)_max_ < 0.001
4516 reflections	Δρ_max_ = 0.43 e Å^−^^3^
242 parameters	Δρ_min_ = −0.30 e Å^−^^3^
Primary atom site location: structure-invariant direct methods	Extinction correction: none

### Special details

**Table:**

Geometry. Bond distances, angles *etc*. have been calculated using the rounded fractional coordinates. All su's are estimated from the variances of the (full) variance-covariance matrix. The cell e.s.d.'s are taken into account in the estimation of distances, angles and torsion angles
Refinement. Refinement on *F*^2^ for ALL reflections except those flagged by the user for potential systematic errors. Weighted *R*-factors *wR* and all goodnesses of fit *S* are based on *F*^2^, conventional *R*-factors *R* are based on *F*, with *F* set to zero for negative *F*^2^. The observed criterion of *F*^2^ > σ(*F*^2^) is used only for calculating -*R*-factor-obs *etc*. and is not relevant to the choice of reflections for refinement. *R*-factors based on *F*^2^ are statistically about twice as large as those based on *F*, and *R*-factors based on ALL data will be even larger.

### Fractional atomic coordinates and isotropic or equivalent isotropic displacement parameters (Å^2^)

**Table:**

	*x*	*y*	*z*	*U*_iso_*/*U*_eq_	
Ni1	0.23220 (2)	0.37068 (3)	0.22317 (1)	0.0317 (1)	
O1	0.26131 (9)	0.2742 (2)	0.33739 (8)	0.0434 (4)	
O2	0.20315 (9)	0.08148 (18)	0.17407 (8)	0.0398 (4)	
N1	0.39913 (11)	0.3396 (2)	0.28785 (9)	0.0334 (4)	
N2	0.38916 (17)	0.2368 (3)	0.47094 (11)	0.0477 (6)	
N3	0.06825 (11)	0.3406 (2)	0.17609 (8)	0.0323 (4)	
N4	0.07207 (16)	−0.1400 (3)	0.09546 (11)	0.0461 (6)	
N5	0.24200 (12)	0.6613 (2)	0.26310 (10)	0.0428 (5)	
N6	0.31950 (13)	0.7291 (2)	0.32014 (10)	0.0385 (5)	
N7	0.39487 (16)	0.8009 (3)	0.37649 (12)	0.0648 (7)	
N8	0.21165 (13)	0.4829 (2)	0.11250 (10)	0.0447 (6)	
N9	0.20157 (13)	0.3801 (2)	0.05837 (10)	0.0423 (5)	
N10	0.1963 (2)	0.2815 (3)	0.00574 (13)	0.0815 (9)	
C1	0.44295 (14)	0.2970 (3)	0.36860 (11)	0.0342 (6)	
C2	0.55345 (15)	0.2892 (3)	0.42221 (13)	0.0468 (7)	
C3	0.62099 (16)	0.3289 (3)	0.39169 (14)	0.0531 (8)	
C4	0.57737 (15)	0.3711 (3)	0.30972 (13)	0.0482 (7)	
C5	0.46602 (15)	0.3750 (3)	0.25924 (12)	0.0403 (6)	
C6	0.35865 (14)	0.2657 (3)	0.39230 (11)	0.0352 (6)	
C7	0.02452 (13)	0.1678 (3)	0.13675 (10)	0.0341 (5)	
C8	−0.08351 (14)	0.1241 (3)	0.10454 (12)	0.0467 (6)	
C9	−0.14833 (16)	0.2628 (4)	0.11367 (13)	0.0573 (8)	
C10	−0.10408 (16)	0.4361 (4)	0.15429 (12)	0.0512 (7)	
C11	0.00463 (15)	0.4720 (3)	0.18414 (11)	0.0426 (6)	
C12	0.10687 (14)	0.0314 (3)	0.13626 (10)	0.0340 (6)	
H2	0.58190	0.25790	0.47780	0.0560*	
H3	0.69590	0.32680	0.42690	0.0640*	
H4	0.62190	0.39680	0.28820	0.0580*	
H5	0.43630	0.40330	0.20330	0.0480*	
H8	−0.11240	0.00400	0.07720	0.0560*	
H9	−0.22170	0.23720	0.09210	0.0690*	
H10	−0.14620	0.52930	0.16190	0.0610*	
H11	0.03440	0.59240	0.21080	0.0510*	
H12	0.4567 (19)	0.229 (3)	0.5093 (14)	0.060 (7)*	
H13	0.3411 (17)	0.224 (3)	0.4844 (12)	0.049 (6)*	
H14	0.1179 (17)	−0.220 (3)	0.1030 (13)	0.048 (7)*	
H15	−0.001 (2)	−0.180 (3)	0.0684 (15)	0.076 (8)*	

### Atomic displacement parameters (Å^2^)

**Table:**

	*U*^11^	*U*^22^	*U*^33^	*U*^12^	*U*^13^	*U*^23^
Ni1	0.0266 (1)	0.0335 (1)	0.0333 (1)	−0.0016 (1)	0.0141 (1)	−0.0003 (1)
O1	0.0295 (7)	0.0589 (9)	0.0385 (7)	−0.0032 (6)	0.0149 (6)	0.0086 (6)
O2	0.0307 (7)	0.0327 (7)	0.0541 (8)	0.0009 (5)	0.0203 (6)	−0.0001 (6)
N1	0.0283 (7)	0.0316 (8)	0.0397 (8)	−0.0026 (6)	0.0170 (6)	−0.0001 (7)
N2	0.0427 (11)	0.0594 (12)	0.0359 (10)	−0.0036 (9)	0.0164 (9)	0.0035 (9)
N3	0.0282 (7)	0.0349 (9)	0.0314 (7)	0.0022 (6)	0.0136 (6)	0.0001 (7)
N4	0.0430 (10)	0.0347 (10)	0.0493 (10)	0.0040 (9)	0.0154 (8)	−0.0012 (8)
N5	0.0387 (9)	0.0392 (10)	0.0429 (9)	−0.0012 (7)	0.0154 (8)	−0.0075 (8)
N6	0.0427 (9)	0.0333 (9)	0.0391 (9)	0.0023 (8)	0.0206 (8)	−0.0010 (8)
N7	0.0520 (12)	0.0594 (12)	0.0527 (11)	−0.0042 (9)	0.0048 (9)	−0.0122 (10)
N8	0.0602 (10)	0.0388 (10)	0.0398 (9)	−0.0011 (8)	0.0290 (8)	−0.0004 (8)
N9	0.0484 (9)	0.0424 (10)	0.0381 (9)	−0.0028 (8)	0.0236 (8)	0.0049 (9)
N10	0.138 (2)	0.0638 (14)	0.0597 (13)	−0.0046 (14)	0.0629 (15)	−0.0130 (11)
C1	0.0309 (9)	0.0304 (10)	0.0373 (10)	−0.0016 (8)	0.0145 (8)	−0.0022 (8)
C2	0.0319 (10)	0.0545 (13)	0.0433 (11)	−0.0021 (9)	0.0114 (9)	−0.0047 (10)
C3	0.0271 (10)	0.0607 (15)	0.0608 (14)	0.0003 (9)	0.0147 (10)	−0.0090 (11)
C4	0.0366 (10)	0.0476 (12)	0.0687 (14)	−0.0008 (10)	0.0329 (10)	−0.0020 (11)
C5	0.0390 (10)	0.0381 (11)	0.0501 (11)	−0.0008 (9)	0.0274 (9)	0.0006 (10)
C6	0.0341 (10)	0.0321 (11)	0.0351 (10)	−0.0031 (8)	0.0146 (8)	0.0016 (8)
C7	0.0283 (8)	0.0435 (11)	0.0275 (8)	0.0007 (8)	0.0121 (7)	0.0025 (8)
C8	0.0320 (10)	0.0598 (13)	0.0411 (10)	−0.0076 (10)	0.0134 (8)	−0.0059 (10)
C9	0.0258 (10)	0.097 (2)	0.0460 (12)	0.0026 (11)	0.0162 (9)	−0.0008 (12)
C10	0.0369 (11)	0.0722 (16)	0.0451 (11)	0.0160 (11)	0.0214 (10)	0.0006 (11)
C11	0.0411 (10)	0.0473 (12)	0.0392 (10)	0.0116 (9)	0.0204 (9)	0.0022 (9)
C12	0.0360 (10)	0.0320 (10)	0.0317 (9)	0.0003 (8)	0.0157 (8)	0.0024 (8)

### Geometric parameters (Å, °)

**Table:**

Ni1—O1	2.0701 (13)	N4—H14	0.80 (2)
Ni1—O2	2.0941 (12)	N4—H15	0.94 (3)
Ni1—N1	2.0685 (17)	C1—C6	1.502 (3)
Ni1—N3	2.0559 (17)	C1—C2	1.378 (3)
Ni1—N5	2.0652 (14)	C2—C3	1.381 (4)
Ni1—N8	2.0863 (17)	C3—C4	1.364 (3)
O1—C6	1.243 (2)	C4—C5	1.379 (3)
O2—C12	1.234 (3)	C7—C8	1.376 (3)
N1—C1	1.344 (2)	C7—C12	1.497 (3)
N1—C5	1.339 (3)	C8—C9	1.386 (3)
N2—C6	1.324 (3)	C9—C10	1.356 (4)
N3—C7	1.344 (2)	C10—C11	1.382 (3)
N3—C11	1.331 (3)	C2—H2	0.9300
N4—C12	1.328 (3)	C3—H3	0.9300
N5—N6	1.172 (2)	C4—H4	0.9300
N6—N7	1.163 (3)	C5—H5	0.9300
N8—N9	1.175 (2)	C8—H8	0.9300
N9—N10	1.159 (3)	C9—H9	0.9300
N2—H12	0.86 (3)	C10—H10	0.9300
N2—H13	0.85 (3)	C11—H11	0.9300
			
O1—Ni1—O2	93.30 (5)	C2—C1—C6	125.28 (17)
O1—Ni1—N1	78.45 (6)	C1—C2—C3	118.6 (2)
O1—Ni1—N3	89.76 (6)	C2—C3—C4	119.7 (2)
O1—Ni1—N5	88.69 (6)	C3—C4—C5	118.8 (2)
O1—Ni1—N8	175.91 (6)	N1—C5—C4	122.39 (18)
O2—Ni1—N1	94.34 (6)	O1—C6—N2	121.6 (2)
O2—Ni1—N3	78.08 (6)	N2—C6—C1	119.7 (2)
O2—Ni1—N5	173.44 (7)	O1—C6—C1	118.71 (16)
O2—Ni1—N8	89.94 (5)	C8—C7—C12	125.59 (18)
N1—Ni1—N3	165.67 (6)	N3—C7—C12	112.41 (17)
N1—Ni1—N5	92.18 (6)	N3—C7—C8	121.95 (19)
N1—Ni1—N8	98.83 (7)	C7—C8—C9	118.6 (2)
N3—Ni1—N5	95.69 (7)	C8—C9—C10	119.6 (2)
N3—Ni1—N8	93.36 (7)	C9—C10—C11	118.9 (2)
N5—Ni1—N8	88.36 (6)	N3—C11—C10	122.39 (19)
Ni1—O1—C6	114.90 (13)	N4—C12—C7	117.8 (2)
Ni1—O2—C12	114.68 (13)	O2—C12—N4	123.2 (2)
Ni1—N1—C1	114.71 (14)	O2—C12—C7	119.01 (17)
Ni1—N1—C5	126.71 (13)	C1—C2—H2	121.00
C1—N1—C5	118.28 (18)	C3—C2—H2	121.00
Ni1—N3—C7	115.52 (13)	C2—C3—H3	120.00
Ni1—N3—C11	125.88 (13)	C4—C3—H3	120.00
C7—N3—C11	118.55 (18)	C3—C4—H4	121.00
Ni1—N5—N6	123.67 (13)	C5—C4—H4	121.00
N5—N6—N7	178.2 (2)	N1—C5—H5	119.00
Ni1—N8—N9	122.99 (12)	C4—C5—H5	119.00
N8—N9—N10	176.9 (3)	C7—C8—H8	121.00
H12—N2—H13	119 (2)	C9—C8—H8	121.00
C6—N2—H12	122.0 (18)	C8—C9—H9	120.00
C6—N2—H13	119.4 (14)	C10—C9—H9	120.00
C12—N4—H14	116.3 (16)	C9—C10—H10	121.00
C12—N4—H15	123.0 (15)	C11—C10—H10	121.00
H14—N4—H15	119 (2)	N3—C11—H11	119.00
N1—C1—C2	122.2 (2)	C10—C11—H11	119.00
N1—C1—C6	112.46 (17)		

### Hydrogen-bond geometry (Å, °)

**Table:**

*D*—H···*A*	*D*—H	H···*A*	*D*···*A*	*D*—H···*A*
N2—H12···N7^i^	0.86 (3)	2.12 (2)	2.967 (3)	165 (3)
N2—H13···N10^ii^	0.85 (3)	2.31 (3)	3.154 (4)	172 (2)
N4—H14···N8^iii^	0.80 (2)	2.36 (2)	3.136 (3)	164 (2)
N4—H15···N10^iv^	0.94 (3)	2.50 (3)	3.442 (4)	179 (3)
C2—H2···N7^i^	0.93	2.61	3.516 (3)	163
C4—H4···O2^v^	0.93	2.55	3.318 (3)	140
C8—H8···N10^iv^	0.93	2.37	3.297 (3)	174
C10—H10···O1^vi^	0.93	2.33	3.256 (3)	172

Symmetry codes: (i) −*x*+1, −*y*+1, −*z*+1; (ii) *x*, −*y*+1/2, *z*+1/2; (iii) *x*, *y*−1, *z*; (iv) −*x*, −*y*, −*z*; (v) −*x*+1, *y*+1/2, −*z*+1/2; (vi) −*x*, *y*+1/2, −*z*+1/2.
